# An Improved Variational Bayesian-Based Adaptive Federated Kalman Filter for Multi-Sensor Integrated Navigation Systems

**DOI:** 10.3390/s25237173

**Published:** 2025-11-24

**Authors:** Yuwei Yan, Jing Yang

**Affiliations:** School of Automation Science and Electrical Engineering, Beihang University, Beijing 100191, China; zy2203118@buaa.edu.cn

**Keywords:** information fusion, integrated navigation, federated Kalman filter, variational Bayesian, adaptive filter

## Abstract

Efficient fusion of navigation sensor data with different output frequencies and data types is critical for ensuring that vehicle-mounted integrated navigation systems consistently provide stable, reliable navigation solutions in complex dynamic operational environments. To address the degradation of estimation accuracy caused by the noise characteristics mismatch of sensor measurement, an information fusion framework based on federated Kalman filter (FKF) framework is designed by incorporating an improved variational Bayesian-based adaptive Kalman filter (IVBAKF) as the core estimation module of local filters. IVBAKF mitigates the impact of uncertain measurement noise from navigation sensors through effectively estimating the measurement noise covariance matrix (MNCM) by leveraging an adaptive forgetting factor. The adjustment strategy for the forgetting factor employs a predefined mapping function derived from the squared Mahalanobis distance (SMD) of the measurement innovation, which serves as an indicator for detecting anomalies in measurement noise within the FKF, thereby enhancing the tracking capability for the MNCMs. The effectiveness of the proposed algorithm is validated through Monte Carlo simulation-based comparative experiments. The simulation results demonstrate that compared to the FKF-based baseline algorithm with nominal covariance matrices, the proposed algorithm achieves an average reduction of 43.21% in the Root Mean Square Errors (RMSEs) of the estimated navigation parameters in scenarios characterized by uncertain and time-varying measurement noise. Thus, the robustness of the proposed algorithm against complex measurement noise conditions is verified.

## 1. Introduction

High-accuracy, reliable positional information is indispensable for safety-critical systems such as autonomous vehicles, navigation platforms, mobile robots, etc. In these applications, real-time dynamic positioning serves as a core enabling technology, with its performance directly determining operational safety and service efficacy. To achieve robust high-precision solutions of positioning in complex dynamic environments, asynchronous multi-sensor integrated navigation systems have been widely deployed. These systems fuse data from sensors with complementary operating principles, such as inertial navigation system (INS), Global Navigation Satellite System (GNSS), magnetometers (MAG), odometer (ODO), vision sensors, and so on, to construct asynchronous multi-sensor integrated navigation systems to mitigate inherent limitations of individual sensor [1,2,3].

Despite the dominance of deep learning in contemporary research, analytics-based multi-sensor navigation information fusion approaches persist as an indispensable methodological paradigm owing to their inherent interpretability and computational efficiency. Among widely studied representative implementations, filtering-based methods [4,5], graph optimization techniques [6,7], and their hybrid combinations constitute essential technical pathways. Graph optimization-based approaches offer advantages, including global optimality, modeling flexibility, and enhanced robustness, and have become one of the most active research areas in the field. Nevertheless, filtering-based approaches remain the core and fundamental solution for online real-time applications due to their computational efficiency, low resource requirements, minimal complexity, and solid theoretical foundation [8].

Compared to centralized filters, decentralized filtering has been widely used in state estimation based on multi-source measurement information due to its flexibility and fault tolerance [9,10,11]. The federated Kalman filter (FKF) [12,13], a prominent embodiment of decentralized filtering with a hierarchical filtering structure, has been widely applied in multi-source navigation information fusion systems. It operates on an information distribution framework between the master filter and local filters. The feedback from the master filter to local filters depends critically on the specific information distribution strategies. Notably, the information distribution coefficient serves as a pivotal parameter, directly determining both the architectural configuration and operational performance of the FKF. Conventional FKF employs fixed Information Sharing Factor (ISF) to partition information among local filters [14]. However, due to complex environmental interference or sensor performance degradation, certain navigation sensors may exhibit time-varying noise characteristics that are difficult to accurately characterize. The resulting mismatch in measurement noise characteristics within the FKFs will inevitably degrade estimation accuracy, consequently deteriorating the integrated navigation system’s performance. To address this challenge, significant research efforts have been devoted to developing adaptive processing mechanisms for the FKF at distinct implementation levels.

A widely adopted adaptive processing mechanism is dynamically adjusting the ISFs based on the quantitative performance evaluation of local filters within the FKF framework [15,16,17]. These performance metrics are typically derived from innovation sequences or covariance matrices associated with prior/posterior state estimates of local filters. Ref. [18] presents a fault-tolerant integrated navigation scheme for GNSS/SINS/DVL/CNS systems by using adaptive ISFs derived from a simplified state Chi-square test to enhance accuracy and stability in complex environments. Ref. [19] proposes an adaptive FKF with time-varying ISFs based on observability analysis to improve the navigation accuracy of Unmanned Ground Vehicle (UGV) in dynamic environments. Ref. [20] proposes adaptive FKF by adjusting the ISF according to the error covariance matrix, residual, trace, and other information of each local filter. The above-mentioned methods maintain the estimation accuracy of FKF by adjusting the information fusion weights of local filters, but fail to mitigate the degradation of estimation accuracy in local filters caused by anomalous measurements, especially when only a few navigation sensors are available.

Alternative notable adaptive processing mechanism is replacing the standard Kalman Filter (KF) employed in the local filters of conventional FKF with adaptive filtering [21]. Ref. [22] adopts the strong tracking filter as local filters and adaptively adjusts the gain matrices of these filters by introducing fading factors, thereby enabling timely tracking of state mutations. Multi-sensor optimal data fusion methods based on adaptive Unscented Kalman Filter (UKF) are proposed for INS/GNSS/CNS integrated navigation systems in [23,24] to improve accuracy and adaptability in the presence of process noise and abnormal measurements, respectively. Ref. [25] proposes a federated Sage–Husa adaptive filter for SINS/CNS/GNSS integrated navigation systems with time-varying or mis-estimated state noise. Ref. [26] proposes an adaptive federated filter based on interacting multiple model (IMM) filtering for SINS/GPS/ODO integrated navigation systems to mitigate noise with unknown or randomly varying statistical properties and outliers. In this design, a modified KF leveraging innovation orthogonality serves as the parallel model filter. Ref. [27] presents a nonlinear double model based on the improved federated extended Kalman filter (EKF) for integrated navigation. Although a lot of studies have improved robustness to noise mismatch and outliers in specific application scenarios, significant challenges remain in handling systems with unknown time-varying noise statistics.

Variational Bayesian (VB) approximation has emerged as a promising noise covariance matching technique, garnering significant attention in recent research [28,29,30,31,32,33,34]. Variational Bayesian adaptive Kalman filter (VBAKF) algorithms proposed in [28,29] recursively construct separable approximations of the joint posterior distribution of states and noise parameters at each step of KF. To address simultaneous challenges of unknown measurement noise covariance and outliers, VB-based methods for estimating measurement noise covariance and maximum correntropy criterion (MCC)-based outlier suppression are integrated with KF in [30] and with cubature Kalman filtering (CKF) in [31] to enhance algorithm robustness. Ref. [32] develops a variational Bayesian federated cubature Kalman filter (VBFCKF) for multi-sensor fusion navigation system in complex environments, where the third-order spherical radial cubature rule and variational Bayesian theory are introduced in local filters. Ref. [33] proposes a novel VB-based adaptive federated Kalman filter to jointly estimate the state vector, the one-step prediction error covariance matrix (PECM), and the MNCM under inverse-Wishart (IW) distribution assumption. Ref. [34] proposes an improved variational Bayesian-based adaptive FKF by dynamically adjusting the ISFs according to the accuracy of local filters, which improves navigation reliability and accuracy. These methods enhance the capability to accommodate measurement noise mismatches, and improve the robustness of the FKF algorithm.

To mitigate the impact of measurement noise mismatch on navigation accuracy, an improved variational Bayesian-based adaptive federated Kalman filter (IVBAFKF) for vehicle-mounted asynchronous multi-sensor integrated navigation systems is proposed. The main contributions of this work are as follows: (A) To address the need for computationally efficient fusion of time-varying navigation sources, an FKF framework employing physics-based partitioned local filters is proposed. This design achieves significant reductions in algorithmic complexity to ensure practical implementation feasibility. (B) To improve state estimation accuracy under uncertain measurement noise conditions, an improved VBAKF algorithm is employed for each local filter. (C) To enhance robustness against mismatched measurement noise conditions, a novel forgetting factor adjustment mechanism driven by measurement innovation SMD is designed. (D) The effectiveness of the proposed method was validated through comparative simulations on INS/GNSS/ODO/MAG integrated navigation system.

The rest of this paper is organized as follows: Section 2 establishes the FKF-based fusion framework and the model for multi-sensor integrated navigation systems, capable of handling variable numbers and types of information sources. Section 3 introduces the theoretical foundations of the IVBAFKF algorithm and the proposed adjustment strategy. Section 4 evaluates the performance of the proposed algorithm through simulations. Finally, the conclusions are drawn in Section 5.

## 2. Problem Formulation

### 2.1. Information Fusion Structure of Multi-Sensor Integrated Navigation System

In conventional multi-sensor integrated navigation systems based on the FKF structure, the number of local filters is determined by the number of navigation sensors involved in information fusion. Typically, each local filter consists of the reference Inertial Measurement Unit (IMU) combined and one or more auxiliary navigation sensors. Consequently, the dynamic isolation and reintegration of navigation sensors, triggered by signal degradation or fault, cause the number of navigation sources in the integrated navigation system to change dynamically. For systems with an uncertain number of navigation sources, the number of available sensors participating in navigation dynamically varies. As a result, the number of local filters must be adjusted accordingly, resulting in significant computational complexity and posing challenges for practical engineering implementation.

**Figure 1:**
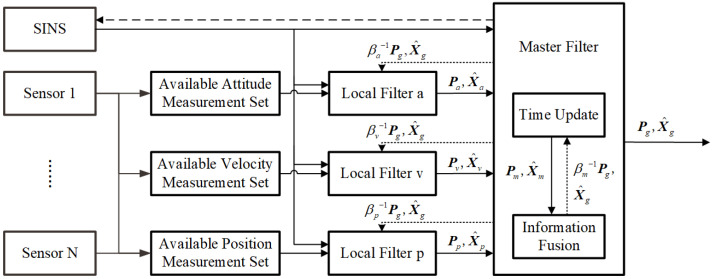
The information fusion framework based on adaptive FKF.

In this paper, an information fusion framework based on FKF is designed to accommodate the uncertain number of navigation sources, as shown in Figure 1. The available sensor measurements are first categorized into subsets based on physical quantities, including attitude, velocity, and position, and processed in parallel through dedicated local filters. Each local filter employs an improved variational Bayesian-based AKF algorithm, replacing conventional KF to mitigate the impact of time-varying sensor-specific noise characteristics. Let i∈{m,a,v,p} denote the master filter and local filters corresponding to attitude, velocity, and position subsets, respectively. The estimation results ${\hat{X}}_{i}$ and $P_{i}$ of filter *i* are subsequently fused to generate the global estimation ${\hat{X}}_{g}$ and $P_{g}$. Meanwhile, the global estimation is used to reset filter *i* periodically according to the ISF $\beta_{i}$ ($\sum_{i}\beta_{i}=1$), to ensure the accuracy of FKF. The dashed line from the master filter to the SINS denotes the use of global estimates to provide feedback for correcting navigation errors. The dashed line from the master filter to the local filter denotes the feedback reset of the local filter using these global estimates.

### 2.2. Sytem Model

The inertial navigation error model and the measurement model of navigation sensors are adopted to establish the system model of FKF shown in Figure 1. This work employs an 18-dimensional state vector composed of a 9-dimensional inertial navigation system (INS) error component and a 9-dimensional inertial sensor error component, structured as follows:

$$X={\left[\begin{array}{cccccc} \phi^{T} & {\delta v}^{T} & {\delta p}^{T} & {\varepsilon_{b}}^{T} & {\varepsilon_{r}}^{T} & {\nabla_{b}}^{T} \end{array}\right]}^{T}$$

where $\phi ={\left[\begin{array}{ccc} \phi_{E} & \phi_{N} & \phi_{U} \end{array}\right]}^{T}$ represents the misalignment angles of INS along the platform axis, and subscripts E, N, and U denote the east, north, and up directions, respectively. $\delta v={\left[\begin{array}{ccc} \delta v_{E} & \delta v_{N} & \delta v_{U} \end{array}\right]}^{T}$ is the velocity error. $\delta p={\left[\begin{array}{ccc} \delta L & \delta \lambda & \delta h \end{array}\right]}^{T}$ is the position error with the element of the latitude error δL, the longitude error δλ, and the altitude error δh. $\varepsilon_{b}$ and $\varepsilon_{r}$ are the constant drift error and slowly varying drift error of the gyroscope, respectively. $\nabla_{b}$ is the zero bias of the accelerometer.

According to [35,36], the master filter and the local filters within the FKF framework utilize the above-mentioned state vector to construct the system’s state equation, defined mathematically as

(1) $${\dot{X}}^{i}=FX^{i}+\mathrm{GW}\left(i=m,a,v,p\right)$$

where $X^{i}$ is the state vector of filter *i*. F is the state transition matrix. G is the process noise transition matrix. $W={\left[\begin{array}{ccc} {w_{g}}^{T} & {w_{rg}}^{T} & {w_{a}}^{T} \end{array}\right]}^{T}$ is the process noise vector, in which $w_{g}$ and $w_{rg}$ are the noise vectors associated with gyroscope random errors and slowly varying drifts respectively, and $w_{a}$ is the noise vector of accelerometer random errors. The process noise is commonly modeled as zero-mean Gaussian white noise.

In this work, both the local filters and the master filter are designed to estimate the same 18-dimensional state vector X. While all the filters estimate an identical set of states, their respective estimations and associated error covariance matrices differ due to the distinct observational updates from their dedicated sensors.

Based on the physical quantity classification criteria, navigation sensor data are allocated to their designated local filters for measurement updates, facilitating real-time dynamic correction of state estimation errors within each filter module. The measurement model for local filter *i* (i=a,v,p) related to sensor *s* can be expressed as

(2) $$Z_{s}^{i}=H_{s}^{i}X^{i}+\nu_{s}^{i}$$

where the observation vector $Z_{s}^{i}$ is constructed by processing physical quantity data *i* from the strapdown INS and sensor *s*. $H_{s}^{i}$ is the measurement matrix. $\nu_{s}^{i}$ is the measurement noise vector. It is commonly modeled as zero-mean Gaussian white noise, $\nu_{s}^{i}∼N\left(0,R_{s}^{i}\right)$, where the measurement noise covariance matrix $R_{s}^{i}$ is fundamentally governed by the noise characteristics of sensor *s* during observation of physical quantity *i*, theoretically. Nevertheless, precise determination of $R_{s}^{i}$ is typically challenging due to disturbances arising from complex measurement environments or degradation in sensor performance.

When redundant measurements of a physical quantity are available from multiple independent navigation sensors, the local filter adopts sequential processing methodology. This scheme enables effective integration of asynchronous sensor measurement while accommodating heterogeneous sampling rates.

Subsequently, the measurement models are established according to the physical quantity types of the observations obtained from the navigation sensors.

- Attitude Measurement Model

The attitude measurement equation, which integrates sensor *s*’s attitude observations $a_{s}$ with the attitude solution from the SINS $a_{sins}$, is formulated as follows:

(3) $$Z_{s}^{a}=a_{sins}-a_{s}=H_{s}^{a}X^{a}+\nu_{s}^{a}$$

where the attitude measurement vector $Z_{s}^{a}$ is obtained from the attitude Euler angle $a_{sins}={\left[\begin{array}{ccc} \gamma_{sins} & \psi_{sins} & \theta_{sins} \end{array}\right]}^{T}$ and $a_{s}={\left[\begin{array}{ccc} \gamma_{s} & \psi_{s} & \theta_{s} \end{array}\right]}^{T}$ denote the roll, yaw, and pitch obtained by SINS and sensor *s*, respectively. $H_{s}^{a}$ is the measurement matrix. $\nu_{s}^{a}$ denotes the attitude measurement noise vector.

Obviously, $Z_{s}^{a}$ represents the error of Euler angles. However, the attitude-related state vector component is the misalignment angle ϕ. Consequently, the measurement matrix $H_{s}^{a}$ is derived from their kinematic relationship and given as follows

$$H_{s}^{a}=\left[\begin{array}{cccccc} M & 0_{3\times 3} & 0_{3\times 3} & 0_{3\times 3} & 0_{3\times 3} & 0_{3\times 3} \end{array}\right]$$

where M is the transformation matrix

$$M=\left[\begin{array}{ccc} -\frac{\sin\psi }{\cos\theta } & -\frac{\cos\psi }{\cos\theta } & 0\\ -\frac{\sin\theta \sin\psi }{\cos\theta } & -\frac{\sin\theta \cos\psi }{\cos\theta } & 1\\ -\cos\psi & \sin\psi & 0 \end{array}\right]$$

- Velocity Measurement Model

The velocity measurement equation, which integrates sensor *s*’s velocity observations $v_{s}$ with the velocity solution from the SINS $v_{sins}$, is expressed as

(4) $$Z_{s}^{v}=v_{sins}-v_{s}=H_{s}^{v}X^{v}+\nu_{s}^{v}$$

where the velocity measurement vector $Z_{s}^{a}$ is obtained from $v_{sins}={\left[\begin{array}{ccc} v_{E,sins} & v_{N,sins} & v_{U,sins} \end{array}\right]}^{T}$ and $v_{s}={\left[\begin{array}{ccc} v_{E,s} & v_{N,s} & v_{U,s} \end{array}\right]}^{T}$. $\nu_{s}^{v}$ is the velocity measurement noise vector. The corresponding measurement matrix $H_{s}^{v}$ is given by

$$H_{s}^{v}=\left[\begin{array}{cccccc} 0_{3\times 3} & I_{3\times 3} & 0_{3\times 3} & 0_{3\times 3} & 0_{3\times 3} & 0_{3\times 3} \end{array}\right]$$

- Position Measurement Model

The position measurement equation, which integrates sensor *s*’s position observations $p_{s}$ with the position solution from the SINS $p_{sins}$, is expressed as

(5) $$Z_{s}^{p}=\left[\begin{array}{c} \left(L_{sins}-L_{s}\right)\left(R_{M}+h_{sins}\right)\\ \left(\lambda_{sins}-\lambda_{s}\right)\left(R_{N}+h_{sins}\right)\cos L_{sins}\\ h_{sins}-h_{s} \end{array}\right]=H_{s}^{p}X^{p}+\nu_{s}^{p}$$

where the position measurement vector $Z_{s}^{p}$ is obtained from $p_{sins}={\left[\begin{array}{ccc} L_{sins} & \lambda_{sins} & h_{sins} \end{array}\right]}^{T}$ and $p_{s}={\left[\begin{array}{ccc} L_{s} & \lambda_{s} & h_{s} \end{array}\right]}^{T}$. $\nu_{s}^{p}$ denotes the position measurement noise vector. $R_{N}$ and $R_{M}$ represent the meridional and prime vertical radii of curvature. The measurement matrix $H_{s}^{p}$ is given by

$$H_{s}^{p}=\left[\begin{array}{cccccccc} 0_{1\times 3} & 0_{1\times 3} & R_{M}+h_{sins} & 0 & 0 & 0_{1\times 3} & 0_{1\times 3} & 0_{1\times 3}\\ 0_{1\times 3} & 0_{1\times 3} & 0 & \left(R_{N}+h_{sins}\right)\cos L_{sins} & 0 & 0_{1\times 3} & 0_{1\times 3} & 0_{1\times 3}\\ 0_{1\times 3} & 0_{1\times 3} & 0 & 0 & 1 & 0_{1\times 3} & 0_{1\times 3} & 0_{1\times 3} \end{array}\right]$$

- Vehicular Navigation System composed of Specific Sensors

This paper introduces the proposed method through a multi-sensor integrated navigation system composed of an IMU, GNSS, odometer, and magnetometer as a representative example. Such a configuration, typically serving as the core module for vehicular navigation systems, provides a practical framework to demonstrate the proposed approach. Furthermore, the proposed framework supports integration of supplementary navigation sensors as required.

The GNSS provides velocity and position measurements. When GNSS signals are available, the velocity and position data are integrated with the SINS within the local filter *v* and local filter *p*, respectively. The corresponding measurement equations are presented in (4) and (5). The magnetometer provides attitude measurements of a vehicle. Its information is integrated with SINS in the local filter *a*, following the same measurement equation form as (3).

For vehicular applications, the odometer is typically mounted on the vehicle wheel to measure the forward speed in the body frame. It conventionally provides velocity or position information. In this study, the velocity-based integration scheme is adopted [36]. According to kinematic principles, a vehicle’s lateral and vertical velocity components asymptotically approach zero during ground motion in the absence of lateral slipping or vertical oscillations. This phenomenon, known as the non-holonomic constraints of vehicle kinematics, enables the formulation of virtual velocity observations for integration into the combined navigation framework. Then, $v_{od}={\left[\begin{array}{ccc} 0 & v_{od} & 0 \end{array}\right]}^{T}$, where $v_{od}$ is obtained by odometer.

(6) $$v_{od}=v+\nu_{od}$$

where *v* is the forward speed, and $\nu_{od}$ is the measurement noise of the odometer.

Taking the effects of odometer installation error and scale factor error into consideration, the velocity in the navigation frame can be determined by the odometer as follows:

(7) $$v_{od}^{n}=C_{b}^{n}K_{od}{\left[\begin{array}{ccc} 0 & v_{od} & 0 \end{array}\right]}^{T}$$

where $C_{b}^{n}$ is the attitude matrix. $K_{od}$ can be calculated by

(8) $$K_{od}=\left(1+K_{err}\right)\cdot \left[\begin{array}{ccc} 0 & \varphi_{err} & 0\\ 0 & 1 & 0\\ 0 & \theta_{err} & 0 \end{array}\right]$$

Here $K_{err}$ is the scale factor error of the odometer, and $\varphi_{err}$ and $\theta_{err}$ are the installation angle errors of the odometer. Accordingly, the velocity measurement equation can be established as follows

(9) $$Z_{od}^{v}=v_{sins}-v_{od}^{n}=H_{od}^{v}X^{v}+\nu_{od}^{n}$$

where the measurement matrix is $H_{od}^{v}=H_{s}^{v}$. The measurement noise vector is $\nu_{od}^{n}=C_{b}^{n}K_{od}\nu_{od}$.

Discretizing the continuous model of integrated navigation system described by (1) and (2) yields

(10) $$\begin{array}{cc} \; & X_{k}^{i}=\Phi_{k,k-1}X_{k-1}^{i}+\Gamma_{k|k-1}W_{k-1}\\ \; & Z_{s,k}^{i}=H_{s,k}^{i}X_{k}^{i}+\nu_{s,k}^{i} \end{array}$$

where, $\Phi_{k,k-1}$ is the state transition matrix from time step (k−1) to *k*, $\Gamma_{k|k-1}$ is the process noise transition matrix.

The mathematical model of the asynchronous multi-sensor integrated navigation system has been established above.

## 3. Improved VBAKF-Based Federated Filter Algorithm

### 3.1. VBAKF Algorithm

The VBAKF algorithm employs the Bayesian inference theory to approximate the joint Probability Density Function (PDF) of the state vector X and unknown parameters θ. Its fundamental principle is to approximate the joint posterior PDF $p\left(X,\theta |Z\right)$ using a conjugate exponential distribution-based PDF $q\left(X,\theta \right)$, where Z is the measurement vector [29].

The estimation of the state vector $X_{k}^{i}$ and the MNCM $R_{s,k}^{i}$ of sensor *s* for local filter *i* at time step *k* based on the variational Bayesian theory can be formulated as the ELBO maximization problem, which is expressed as

(11) $$\begin{array}{cc} \; & \max\;L\left(q\left(X_{k}^{i},R_{s,k}^{i}\right)\right)=\;\int \int \;q\left(X_{k}^{i},R_{s,k}^{i}\right)\log\frac{p\left(X_{k}^{i},R_{s,k}^{i},Z_{s,1:k}^{i}\right)}{q\left(X_{k}^{i},R_{s,k}^{i}\right)}dX_{k}^{i}dR_{s,k}^{i}\\ \; & s.t.\int q\left(X_{k}^{i}\right)dX_{k}^{i}=1;\;\int q\left(R_{s,k}^{i}\right)dR_{s,k}^{i}=1 \end{array}$$

According to [33], the optimal solution satisfies the following equations

(12) $$\log q\left(\varphi \right)=E_{\Xi \left(-\varphi \right)}\left[\log p\left(\Xi ,Z_{s,1:k}^{i}\right)\right]+c_{-\varphi }$$

where $\Xi =\left\{X_{k}^{i},R_{s,k}^{i}\right\}$ denotes the random variable set comprising the state vector $X_{k}^{i}$ and the MNCM $R_{s,k}^{i}$. φ represents the variable in Ξ. The notation $\Xi \left(-\varphi \right)$ denotes the set of all the variables in Ξ excluding φ. Consequently, $E_{\Xi \left(-\varphi \right)}$ represents the expectation with respect to all the variables in Ξ except φ, and $c_{-\varphi }$ represents a constant that is independent of φ.

Using fixed-point iteration to solve Equation (12), the variational distribution $q^{\left(j+1\right)}\left(\varphi \right)$ at the (j+1)-th iteration can be computed from

(13) $$\log q^{\left(j+1\right)}\left(R_{s,k}^{i}\right)=E_{X_{k}^{i}}^{\left(j\right)}\left[\log p\left(X_{k}^{i},R_{s,k}^{i},Z_{s,1:k}^{i}\right)\right]+c_{X_{k}^{i}}$$

(14) $$\log q^{\left(j+1\right)}\left(X_{k}^{i}\right)=E_{R_{s,k}^{i}}^{\left(j\right)}\left[\log p\left(X_{k}^{i},R_{s,k}^{i},Z_{s,1:k}^{i}\right)\right]+c_{R_{s,k}^{i}}$$

Since $X_{k}^{i}$, $R_{s,k}^{i}$ and $Z_{s,1:k}^{i}$ are mutually independent, the joint PDF of them can be written as

(15) $$p\left(X_{k}^{i},R_{s,k}^{i},Z_{s,1:k}^{i}\right)=p\left(Z_{s,k}^{i}|X_{k}^{i},R_{s,k}^{i}\right)p\left(X_{k}^{i}|P_{k|k-1}^{i},Z_{s,1:k-1}^{i}\right)p\left(R_{s,k}^{i}|Z_{s,1:k-1}^{i}\right)p\left(Z_{s,1:k-1}^{i}\right)$$

According to the KF principle, $p\left(X_{k}^{i}|P_{k|k-1}^{i},Z_{s,1:k-1}^{i}\right)$ and $p\left(Z_{s,k}^{i}|X_{k}^{i},R_{s,k}^{i}\right)$ both follow the Gaussian distributions, which can be expressed as

(16) $$\begin{array}{cc} \; & p\left(X_{k}^{i}|P_{k|k-1}^{i},Z_{s,1:k-1}^{i}\right)=N\left(X_{k}^{i};{\hat{X}}_{k|k-1}^{i},P_{k|k-1}^{i}\right)\\ \; & ={\left(2\pi \right)}^{-n/2}{\left|P_{k|k-1}^{i}\right|}^{-1/2}\exp\left(-0.5{\left(X_{k}^{i}-{\hat{X}}_{k|k-1}^{i}\right)}^{T}{\left(P_{k|k-1}^{i}\right)}^{-1}\left(X_{k}^{i}-{\hat{X}}_{k|k-1}^{i}\right)\right) \end{array}$$

(17) $$\begin{array}{cc} \; & p\left(Z_{s,k}^{i}|X_{k}^{i},R_{s,k}^{i}\right)=N\left(Z_{s,k}^{i};H_{s,k}^{i}X_{k}^{i},R_{s,k}^{i}\right)\\ \; & ={\left(2\pi \right)}^{-m/2}{\left|R_{s,k}^{i}\right|}^{-1/2}\exp\left(-0.5{\left(Z_{s,k}^{i}-H_{s,k}^{i}X_{k}^{i}\right)}^{T}{\left(R_{s,k}^{i}\right)}^{-1}\left(Z_{s,k}^{i}-H_{s,k}^{i}X_{k}^{i}\right)\right) \end{array}$$

where *n* and *m* are the dimensions of $X_{k}^{i}$ and $Z_{s,k}^{i}$, respectively.

The inverse-Wishart (IW) distribution is adopted in this paper due to its ability to model the uncertainty covariance matrix $R_{s,k}^{i}$ as a random positive-definite matrix, which aligns with the statistical properties of measurement noise. More specifically, the IW distribution is selected as the basic form for $p\left(R_{s,k}^{i}|Z_{s,1:k-1}^{i}\right)$ to construct the variational distribution $q\left(X_{k}^{i},R_{s,k}^{i}\right)$, thereby deriving the formulas of VBAKF embedded in the proposed federated filtering framework.

If $R_{s,k}^{i}$ follows IW distribution with the DOF of $u_{s,k|k-1}^{i}$ and the ISM of $U_{s,k|k-1}^{i}$, then $p\left(R_{s,k}^{i}|Z_{s,1:k-1}^{i}\right)$ can be expressed as

(18) $$\begin{array}{cc} \; & p\left(R_{s,k}^{i}|Z_{s,1:k-1}^{i}\right)=\mathrm{IW}\left(R_{s,k}^{i};u_{s,k|k-1}^{i},U_{s,k|k-1}^{i}\right)\\ \; & =\frac{{\left|U_{s,k|k-1}^{i}\right|}^{0.5u_{s,k|k-1}^{i}}{\left|R_{s,k}^{i}\right|}^{-0.5\left(u_{s,k|k-1}^{i}+m+1\right)}}{2^{0.5mu_{s,k|k-1}^{i}}\Gamma_{m}\left(0.5u_{s,k|k-1}^{i}\right)}\cdot \exp\left(-0.5tr\left(U_{s,k|k-1}^{i}{\left(R_{s,k}^{i}\right)}^{-1}\right)\right) \end{array}$$

where *m* is the dimension of $R_{s,k}^{i}$.

By substituting (16)–(18) into (15), it can be obtained that

(19) $$\begin{array}{cc} \; & p\left(X_{k}^{i},R_{s,k}^{i},Z_{s,1:k}^{i}\right)=N\left(X_{k}^{i};{\hat{X}}_{k|k-1}^{i},P_{k|k-1}^{i}\right)N\left(Z_{s,k}^{i};H_{s,k}^{i}X_{k}^{i},R_{s,k}^{i}\right)\cdot \\ \; & \;\mathrm{IW}\left(R_{s,k}^{i};u_{s,k|k-1}^{i},U_{s,k|k-1}^{i}\right)p\left(Z_{s,1:k-1}^{i}\right) \end{array}$$

Accordingly, $\log p\left(X_{k}^{i},R_{s,k}^{i},Z_{s,1:k}^{i}\right)$ can be written as

(20) $$\begin{array}{cc} \; & \log p\left(X_{k}^{i},R_{s,k}^{i},Z_{s,1:k}^{i}\right)=-0.5\left(u_{s,k|k-1}^{i}+m+2\right)\log\left|R_{s,k}^{i}\right|-0.5tr\left(U_{s,k|k-1}^{i}{\left(R_{s,k}^{i}\right)}^{-1}\right)-\\ \; & \;\;\;\;\;0.5{\left(\epsilon_{s,k}^{i}\right)}^{T}{\left(R_{s,k}^{i}\right)}^{-1}\epsilon_{s,k}^{i}-0.5{\left(\Delta {\tilde{X}}_{k|k-1}^{i}\right)}^{T}{\left(P_{k|k-1}^{i}\right)}^{-1}\Delta {\tilde{X}}_{k|k-1}^{i}+c \end{array}$$

where, $\epsilon_{s,k}^{i}=Z_{s,k}^{i}-H_{s,k}^{i}X_{k}^{i}$, $\Delta {\tilde{X}}_{k|k-1}^{i}=X_{k}^{i}-{\hat{X}}_{k|k-1}^{i}$, *c* is the irrelevant constant.

- MNCM Update

Substituting (20) into (13) yields

(21) $$\begin{array}{cc} \log q^{\left(j+1\right)}\left(R_{s,k}^{i}\right)= & -0.5\left(u_{s,k|k-1}^{i}+m+2\right)\log\left|R_{s,k}^{i}\right|-\\ \; & 0.5\;tr\left(\left(U_{s,k|k-1}^{i}+B_{s,k}^{i,(j)}\right){\left(R_{s,k}^{i}\right)}^{-1}\right)+c_{R_{s,k}^{i}} \end{array}$$

where $c_{R_{s,k}^{i}}$ is a constant independent of $R_{s,k}^{i}$, and $B_{s,k}^{i,(j)}$ can be expressed as

(22) $$\begin{array}{c} B_{s,k}^{i,(j)}=E^{\left(j\right)}\left(\epsilon_{s,k}^{i}{\left(\epsilon_{s,k}^{i}\right)}^{T}\right)=H_{s,k}^{i}P_{k}^{i,(j)}{\left(H_{s,k}^{i}\right)}^{T}+\left(Z_{s,k}^{i}-H_{s,k}^{i}{\hat{X}}_{k}^{i,(j)}\right){\left(Z_{s,k}^{i}-H_{s,k}^{i}{\hat{X}}_{k}^{i,(j)}\right)}^{T} \end{array}$$

Based on the conjugate properties of exponential distributions, $q^{\left(j+1\right)}\left(R_{s,k}^{i}\right)$ theoretically follows IW distribution. Therefore, $q^{\left(j+1\right)}\left(R_{s,k}^{i}\right)=\mathrm{IW}\left(R_{s,k}^{i};u_{s,k}^{i,(j+1)},U_{s,k}^{i,(j+1)}\right)$, and its logarithmic form is

(23) $$\begin{array}{c} \log q^{\left(j+1\right)}\left(R_{s,k}^{i}\right)=-0.5\left(u_{s,k}^{i,(j+1)}+m+1\right)\log\left|R_{s,k}^{i}\right|-0.5\;\mathrm{tr}\left(U_{s,k}^{i,(j+1)}{\left(R_{s,k}^{i}\right)}^{-1}\right)+{c^{\prime }}_{R_{s,k}^{i}} \end{array}$$

where ${c^{\prime }}_{R_{s,k}^{i}}$ is a constant independent of $R_{s,k}^{i}$.

By comparing (21) with (23), $u_{s,k}^{i}$ and $U_{s,k}^{i}$ in the (j+1)-th iteration can be obtained as

(24) $$u_{s,k}^{i,(j+1)}=u_{s,k|k-1}^{i}+1$$

(25) $$U_{s,k}^{i,(j+1)}=U_{s,k|k-1}^{i}+B_{s,k}^{i,(j)}$$

The expectation associated with $R_{s,k}^{i}$ in the (j+1)-th iteration can be expressed as

(26) $$E^{\left(j+1\right)}\left({\left(R_{s,k}^{i}\right)}^{-1}\right)=\left(u_{s,k}^{i,(j+1)}-m-1\right){\left(U_{s,k}^{i,(j+1)}\right)}^{-1}$$

From the above equation, the estimation of $R_{s,k}^{i}$ in the (j+1)-th iteration is given by

(27) $${\hat{R}}_{s,k}^{i,(j+1)}={\left(E^{\left(j+1\right)}\left({\left(R_{s,k}^{i}\right)}^{-1}\right)\right)}^{-1}$$

Since $p\left(R_{s,k}^{i}|Z_{s,1:k-1}^{i}\right)=\mathrm{IW}\left(R_{s,k}^{i};u_{s,k|k-1}^{i},U_{s,k|k-1}^{i}\right)$, when $E\left(R_{s,k}^{i}\right)$ is set as the nominal MNCM, then ${\hat{R}}_{s,k}^{i,(j+1)}$ can be expressed as

(28) $$\begin{array}{cc} {\hat{R}}_{s,k}^{i,(j+1)} & =\frac{U_{s,k}^{i,(j+1)}}{u_{s,k}^{i,(j+1)}-m-1}=\frac{U_{s,k|k-1}^{i}+B_{s,k}^{i,(j)}}{u_{s,k|k-1}^{i}-m}=\frac{\tau }{\tau +1}{\tilde{R}}_{s,k}^{i}+\frac{1}{\tau +1}B_{s,k}^{i,(j)} \end{array}$$

It can be seen that the tuning parameter τ serves to adjust the fusion weights between the prior MNCN ${\tilde{R}}_{s,k}^{i}$ and the iterative estimation result $B_{s,k}^{i,(j)}$. Since $R_{s,k}^{i}$ typically does not undergo abrupt changes within short time intervals, the forgetting factor ρ is introduced for propagating the prior distribution parameters as follows

(29) $$u_{s,k|k-1}^{i}=\rho \left(u_{s,k-1}^{i}-m-1\right)+m+1$$

(30) $$U_{s,k|k-1}^{i}=\rho U_{s,k-1}^{i}$$

The prior MNCM $R_{s}^{i}$ at the initial time is defined according to the IW distribution as follows:

(31) $$p\left(R_{s,0}^{i}\right)=\mathrm{IW}\left(R_{s,0}^{i};u_{s,0}^{i},U_{s,0}^{i}\right)$$

Let $E\left(R_{s,0}^{i}\right)={\tilde{R}}_{s,0}^{i}$, then

(32) $$u_{s,0}^{i}=\tau +m+1$$

(33) $$U_{s,0}^{i}=\tau {\tilde{R}}_{s,0}^{i}$$

- State Vector Update

Substituting (20) into (14) yields

(34) $$\begin{array}{cc} \; & \log q^{\left(j+1\right)}\left(X_{k}^{i}\right)=-0.5{\left(\epsilon_{s,k}^{i}\right)}^{T}E^{\left(j+1\right)}\left({\left(R_{s,k}^{i}\right)}^{-1}\right)\epsilon_{s,k}^{i}-\\ \; & \;\;\;\;0.5{\left(\Delta {\tilde{X}}_{k|k-1}^{i}\right)}^{T}{\left(P_{k|k-1}^{i}\right)}^{-1}\Delta {\tilde{X}}_{k|k-1}^{i}+c_{X_{k}^{i}} \end{array}$$

where, $c_{X_{k}^{i}}$ is a constant independent of $X_{k}^{i}$.

In the (j + 1)th iteration, $p\left(X_{k}^{i}|Z_{s,1:k-1}^{i}\right)$ and $p\left(Z_{s,k}^{i}|X_{k}^{i}\right)$ are defined as

(35) $$p^{\left(j+1\right)}\left(X_{k}^{i}|Z_{s,1:k-1}^{i}\right)=N\left(X_{k}^{i};{\hat{X}}_{k|k-1}^{i},P_{k|k-1}^{i}\right)$$

(36) $$p^{\left(j+1\right)}\left(Z_{s,k}^{i}|X_{k}^{i}\right)=N\left(Z_{s,k}^{i};H_{s,k}^{i}X_{k}^{i},{\hat{R}}_{s,k}^{i,(j+1)}\right)$$

Substituting (27), (35) and (36) into (34) yields

(37) $$q^{\left(j+1\right)}\left(X_{k}^{i}\right)=\frac{1}{c_{k}^{i,(j+1)}}p^{\left(j+1\right)}\left(X_{k}^{i}|Z_{s,1:k-1}^{i}\right)p^{\left(j+1\right)}\left(Z_{s,k}^{i}|X_{k}^{i}\right)$$

where the normalization constant $c_{k}^{i,(j+1)}$ is given by

(38) $$c_{k}^{i,(j+1)}=\int p^{\left(j+1\right)}\left(X_{k}^{i}|Z_{s,1:k-1}^{i}\right)p^{\left(j+1\right)}\left(Z_{s,k}^{i}|X_{k}^{i}\right)dX_{k}^{i}$$

Combining (35)–(38), the posterior PDF $q^{\left(j+1\right)}\left(X_{k}^{i}\right)$ follows the normal distribution

(39) $$q^{\left(j+1\right)}\left(X_{k}^{i}\right)=N\left(X_{k}^{i};{\hat{X}}_{k}^{i,(j+1)},P_{k}^{i,(j+1)}\right)$$

According to [32], ${\hat{X}}_{k}^{i,(j+1)}$ and $P_{k}^{i,(j+1)}$ can be obtained by maximizing (34)

(40) $$K_{s,k}^{i,(j+1)}=P_{k|k-1}^{i}{\left(H_{s,k}^{i}\right)}^{T}{\left(H_{s,k}^{i}P_{k|k-1}^{i}{\left(H_{s,k}^{i}\right)}^{T}+{\hat{R}}_{s,k}^{i,(j+1)}\right)}^{-1}$$

(41) $$P_{k}^{i,(j+1)}=\left(I-K_{s,k}^{i,(j+1)}H_{s,k}^{i}\right)P_{k|k-1}^{i}$$

(42) $${\hat{X}}_{k}^{i,(j+1)}={\hat{X}}_{k|k-1}^{i}+K_{s,k}^{i,(j+1)}\left(Z_{s,k}^{i}-H_{s,k}^{i}{\hat{X}}_{k|k-1}^{i}\right)$$

The iteration number *N* is usually chosen as a sufficiently large constant. After *N* iterations, the approximate posterior PDF is expressed as

(43) $$q\left(X_{k}^{i}\right)=N\left(X_{k}^{i};{\hat{X}}_{k}^{i},P_{k}^{i}\right)\approx q^{\left(N\right)}\left(X_{k}^{i}\right)=N\left(X_{k}^{i};{\hat{X}}_{k}^{i,(N)},P_{k}^{i,(N)}\right)$$

(44) $$q\left(R_{s,k}^{i}\right)=\mathrm{IW}\left(R_{s,k}^{i};u_{s,k}^{i},U_{s,k}^{i}\right)\approx q^{\left(N\right)}\left(R_{s,k}^{i}\right)=\mathrm{IW}\left(R_{s,k}^{i};u_{s,k}^{i,(N)},U_{s,k}^{i,(N)}\right)$$

At time step *k*, the federated filter updates and propagates the local state estimate ${\hat{X}}_{k}^{i}$, error covariance matrix $P_{k}^{i}$ and measurement noise covariance estimate ${\hat{R}}_{s,k}^{i}$ for local filter *i* (i=a,v,p) in accordance with the following equations: ${\hat{X}}_{k}^{i}={\hat{X}}_{k}^{i,(N)}$, ${\hat{X}}_{k}^{i}={\hat{X}}_{k}^{i,(N)}$, ${\hat{R}}_{s,k}^{i}={\hat{R}}_{s,k}^{i,(N)}$.

Additionally, the iteration can be terminated when the following convergence condition is met

(45) $$\frac{∥{\hat{X}}_{k}^{i,(j+1)}-{\hat{X}}_{k}^{i,(j)}∥}{∥{\hat{X}}_{k}^{i,(j+1)}∥}<\varepsilon$$

where ε is the iteration termination threshold, which is set as $\varepsilon =10^{-6}$.

### 3.2. Adaptive Adjustment Strategy for the Forgetting Factor

The following discussion focuses on the influence of the forgetting factor on the estimation accuracy of VBAKF.

Substituting (24)–(26), (29) and (30) into (27) yields

(46) $${\hat{R}}_{s,k}^{i,(j+1)}=\frac{\rho U_{s,k-1}^{i}+B_{s,k}^{i,(j)}}{\rho (u_{s,k-1}^{i}-m-1)+1}$$

According to (44), the estimated MNCM ${\hat{R}}_{s,k-1}^{i}$ is

(47) $${\hat{R}}_{s,k-1}^{i}=\frac{U_{s,k-1}^{i}}{u_{s,k-1}^{i}-m-1}$$

Substituting (47) into (46) yields

(48) $${\hat{R}}_{s,k}^{i,(j+1)}=\frac{\rho (u_{s,k-1}^{i}-m-1)}{\rho (u_{s,k-1}^{i}-m-1)+1}{\hat{R}}_{s,k-1}^{i}+\frac{1}{\rho (u_{s,k-1}^{i}-m-1)+1}B_{s,k}^{i,(j)}$$

Based on (24), (29), and (44), $u_{s,k}^{i}$ is derived as

(49) $$u_{s,k}^{i}-m-1=\rho \left(u_{s,k-1}^{i}-m-1\right)+1$$

Consequently, $u_{s,k-1}^{i}$ can be expressed as

(50) $$u_{s,k-1}^{i}-m-1=\rho^{k-1}\left(u_{s,0}^{i}-m-1\right)+\frac{1-\rho^{k-1}}{1-\rho }$$

Combining (32), (48), and (50) yields

(51) $${\hat{R}}_{s,k}^{i,(j+1)}=\frac{\omega \left(\rho ,\tau ,k\right)}{\omega \left(\rho ,\tau ,k\right)+1}{\hat{R}}_{s,k-1}^{i}+\frac{1}{\omega \left(\rho ,\tau ,k\right)+1}B_{s,k}^{i,(j)}$$

where $\omega \left(\rho ,\tau ,k\right)$ is expressed as

(52) $$\omega \left(\rho ,\tau ,k\right)=\tau \rho^{k}+\frac{\rho -\rho^{k}}{1-\rho }$$

Given that $\rho \in \left(0,1\right]$, if k→+∞, then

(53) $${\hat{R}}_{s,k}^{i,(j+1)}=\rho {\hat{R}}_{s,k-1}^{i}+\left(1-\rho \right)B_{s,k}^{i,(j)}$$

The tuning parameter τ is typically set as a small positive integer. In this case, its influence on ω can be ignored. Therefore, this paper primarily focus on investigating the impact of the forgetting factor ρ on the estimation results.

The forgetting factor ρ in VBAKF is designed to dynamically balance the influence between the prior information ${\hat{R}}_{s,k-1}^{i}$ and the iteratively estimated parameters $B_{s,k}^{i,(j)}$ on the estimation results ${\hat{R}}_{s,k}^{i}$, thereby adapting to varying measurement noise characteristics across different scenarios. When the measurement noise characteristics undergo abrupt variations, a smaller forgetting factor should be selected to reduce the influence of prior information on estimation results. When the measurement noise characteristics change gradually, a larger forgetting factor may be adopted to make the system rely more on prior information, thus reducing the impact of measurement noise on the estimation results.

The VBAKF algorithm rely on the predefined forgetting factor to propagate $u_{s,k}^{i}$ and $U_{s,k}^{i}$. However, this approach often underperforms in complex environments. The adoption of an adaptive forgetting factor is one of the effective approaches to enhance the algorithm’s adaptability [37]. Considering that the measurement innovation can reflect the variation trend of measurement noise, the adaptive adjustment strategy of the forgetting factor is designed based on the SMD of the measurement innovation in this paper.

The SMD of the measurement innovation is calculated by

(54) $$d_{s,k}^{i}={\left(\epsilon_{s,k|k-1}^{i}\right)}^{T}{\left(H_{s,k}^{i}P_{k|k-1}^{i}{\left(H_{s,k}^{i}\right)}^{T}+{\hat{R}}_{s,k}^{i}\right)}^{-1}\epsilon_{s,k|k-1}^{i}$$

where $\epsilon_{s,k|k-1}^{i}$ is the measurement innovation of local filter *i* (i=a,v,p) with respect to sensor *s* at time step *k*. It can be obtained as follows:

(55) $$\epsilon_{s,k|k-1}^{i}=Z_{s,k}^{i}-H_{s,k}^{i}{\hat{X}}_{k|k-1}^{i}$$

When the SMD of the measurement innovation $d_{s,k}^{i}$ is relatively large, which indicates a significant deviation between the observed measurement and their theoretical prediction obtained from the one-step predicted state. This deviation suggests potential model mismatch or the presence of measurement outliers. Thus the forgetting factor should be appropriately reduced to make the algorithm more sensitive to changes in measurement noise characteristics. Conversely, when $d_{s,k}^{i}$ is relatively small, the predefined MNCM can accurately characterize the measurement noise characteristics. In this case, the forgetting factor should be increased to mitigate the influence of measurement noise.

Accordingly, the relationship between the forgetting factor and the SMD of the measurement innovation is constructed as follows

(56) $$\rho_{s,k+1}^{i}=l_{1}+l_{3}\left[1-\tanh\left(l_{2}d_{s,k}^{i}\right)\right]$$

where $l_{1}$, $l_{2}$, and $l_{3}$ are tunable parameters that modulate the dynamic behavior and tracking agility of the forgetting factor. $l_{1}$ sets its lower bound, $l_{2}$ defines its variation range, and $l_{3}$ controls the sensitivity of its adaptation to the SMD of the measurement innovation. It can be observed that, when $d_{s,k}^{i}\to +\infty$, $\rho_{s,k+1}^{i}\to l_{1}$. When $d_{s,k}^{i}\to 0$, $\rho_{s,k+1}^{i}\to l_{1}+l_{3}$. Therefore, the proper selection of $l_{1}$, $l_{2}$, and $l_{3}$ is crucial to ensuring the estimation accuracy of IVBAKF.

In summary, the single-step implementation for sensor *s* in local filter *i* (i=a,v,p) is illustrated in Algorithm 1. If sensor *s* provides valid observations to local filter *i*, the VB-based measurement update is implemented. Otherwise, only the time update is executed.

The estimation results from all filters in FKF are fused as follows

(57) $$P_{k}^{g}={\left[\sum_{i=a,v,p}{\left(P_{k}^{i}\right)}^{-1}+{\left(P_{k}^{m}\right)}^{-1}\right]}^{-1}$$

(58) $${\hat{X}}_{k}^{g}=P_{k}^{g}\left[\sum_{i=a,v,p}{\left(P_{k}^{i}\right)}^{-1}{\hat{X}}_{k}^{i}+{\left(P_{k}^{m}\right)}^{-1}{\hat{X}}_{k}^{m}\right]$$

where ${\hat{X}}_{k}^{m}$ and $P_{k}^{m}$ are the state and its covariance estimated by the master filter, and ${\hat{X}}_{k}^{g}$ and $P_{k}^{g}$ are the outputs of FKF.

The information distribution and reset operations of the *i*-th filter is periodically performed as follows:

(59) $$P_{k}^{i}=\beta_{i}^{-1}P_{k}^{g}$$

(60) $${\hat{X}}_{k}^{i}={\hat{X}}_{k}^{g}$$

**Algorithm 1:** Flowchart of the IVBAFKF algorithm.**Input: **${\hat{X}}_{k-1}^{i}$, $P_{k-1}^{i}$, $u_{s,k-1}^{i}$, $U_{s,k-1}^{i}$, $\Phi_{k|k-1}$, $\Gamma_{k|k-1}$, $Q_{k-1}^{i}$, $H_{s,k}^{i}$, $Z_{s,k}^{i}$, $\rho_{s,k}^{i}$, $l_{1}$, $l_{2}$, $l_{3}$, *m*, τ, *N***Time Update:** ${\hat{X}}_{k|k-1}^{i}=\Phi_{k|k-1}{\hat{X}}_{k-1}^{i}$ $P_{k|k-1}^{i}=\Phi_{k|k-1}P_{k-1}^{i}{\left(\Phi_{k|k-1}\right)}^{T}+\Gamma_{k|k-1}Q_{k-1}^{i}{\left(\Gamma_{k|k-1}\right)}^{T}$**Measurement Update of Variational Bayesian:** **Initialize:**  ${\hat{X}}_{k}^{i,(0)}={\hat{X}}_{k|k-1}^{i}$, $P_{k}^{i,(0)}=P_{k|k-1}^{i}$  $u_{s,k|k-1}^{i}=\rho_{s,k}^{i}\left(u_{s,k-1}^{i}-m-1\right)+m+1$, $U_{s,k|k-1}^{i}=\rho_{s,k}^{i}U_{s,k-1}^{i}$ for j=0:N−1  **Update $q^{\left(j+1\right)}\left(R_{s,k}^{i}\right)=\mathrm{IW}\left(R_{s,k}^{i};u_{s,k}^{i,(j+1)},U_{s,k}^{i,(j+1)}\right)$:**   $B_{s,k}^{i,(j)}=H_{s,k}^{i}P_{k}^{i,(j)}{\left(H_{s,k}^{i}\right)}^{T}+\left(Z_{s,k}^{i}-H_{s,k}^{i}{\hat{X}}_{k}^{i,(j)}\right){\left(Z_{s,k}^{i}-H_{s,k}^{i}{\hat{X}}_{k}^{i,(j)}\right)}^{T}$   $u_{s,k}^{i,(j+1)}=u_{s,k|k-1}^{i}+1$, $U_{s,k}^{i,(j+1)}=U_{s,k|k-1}^{i}+B_{s,k}^{i,(j)}$  **Update $q^{\left(j+1\right)}\left(X_{k}^{i}\right)=N\left(X_{k}^{i};{\hat{X}}_{k}^{i,(j+1)},P_{k}^{i,(j+1)}\right)$:**   ${\hat{R}}_{s,k}^{i,(j+1)}={\left(u_{s,k}^{i,(j+1)}-m-1\right)}^{-1}U_{s,k}^{i,(j+1)}$   $K_{s,k}^{i,(j+1)}=P_{k|k-1}^{i}{\left(H_{s,k}^{i}\right)}^{T}{\left(H_{s,k}^{i}P_{k|k-1}^{i}{\left(H_{s,k}^{i}\right)}^{T}+{\hat{R}}_{s,k}^{i,(j+1)}\right)}^{-1}$   $P_{k}^{i,(j+1)}=\left(I-K_{s,k}^{i,(j+1)}H_{s,k}^{i}\right)P_{k|k-1}^{i}$   ${\hat{X}}_{k}^{i,(j+1)}={\hat{X}}_{k|k-1}^{i}+K_{s,k}^{i,(j+1)}\left(Z_{s,k}^{i}-H_{s,k}^{i}{\hat{X}}_{k|k-1}^{i}\right)$  end for  ${\hat{X}}_{k}^{i}={\hat{X}}_{k}^{i,(N)}$, $P_{k}^{i}=P_{k}^{i,(N)}$  $u_{s,k}^{i}=u_{s,k}^{i,(N)}$, $U_{s,k}^{i}=U_{s,k}^{i,(N)}$, ${\hat{R}}_{s,k}^{i}={\hat{R}}_{s,k}^{i,(N)}$  **Update $\rho_{s,k+1}^{i}$:**   $\epsilon_{s,k|k-1}^{i}=Z_{s,k}^{i}-H_{s,k}^{i}{\hat{X}}_{k|k-1}^{i}$   $d_{s,k}^{i}={\left(\epsilon_{s,k|k-1}^{i}\right)}^{T}{\left(H_{s,k}^{i}P_{k|k-1}^{i}{\left(H_{s,k}^{i}\right)}^{T}+{\hat{R}}_{s,k}^{i}\right)}^{-1}\epsilon_{s,k|k-1}^{i}$   $\rho_{s,k+1}^{i}=l_{1}+l_{3}\left[1-\tanh\left(l_{2}d_{s,k}^{i}\right)\right]$**Output: **${\hat{X}}_{k}^{i}$, $P_{k}^{i}$, $u_{s,k}^{i}$, $U_{s,k}^{i}$, $\rho_{s,k+1}^{i}$

## 4. Simulations

**Figure 2:**
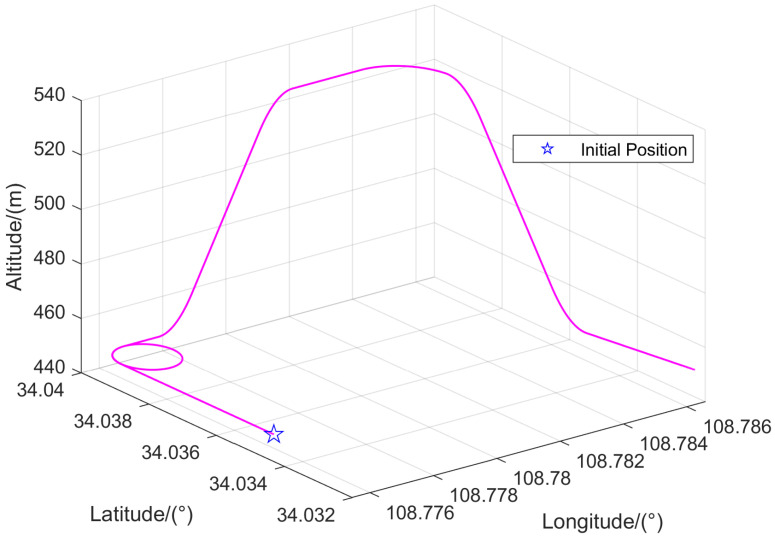
The trajectory generated by the PSINS toolbox.

To evaluate the performance of the proposed IVBAFKF designed for multi-sensor integrated navigation system, simulations are conducted in this section by using the sensor parameters specified in Table 1. A 150 s trajectory generated by the PSINS toolbox in [38] as shown in Figure 2 is employed to emulate a typical urban navigation scenario. The simulation trajectory is set with a starting point at longitude 108.7754° E, latitude 34.0343° N, and altitude 450 m. It primarily includes maneuvers such as uniformly accelerated linear motion, turning, and climbing, with a horizontal position change of approximately 1000 m and a relative altitude variation of about 100 m.

For algorithm performance evaluation, the Averaged RMSE (ARMSE) of state estimations are defined as

(61) $${\mathrm{ARMSE}}_{*}=\sqrt{\frac{1}{TM}\sum_{j=1}^{M}\sum_{k=1}^{T}{∥x_{*,k}-{\hat{x}}_{*,k}^{j}∥}_{2}^{2}}$$

where *T* denotes the number of filtering steps in a single simulation run. *M* denotes the Monte Carlo runs. $x_{*,k}$ and ${\hat{x}}_{*,k}^{j}$(∗=a,v,p) are the reference values and the estimation results of attitude, velocity, and position states at time step *k* during the *j*-th Monte Carlo simulation run, respectively.

To evaluate the effectiveness of the IVBAFKF algorithm with adaptive forgetting factors under dynamic noise conditions, a time-varying measurement noise characteristic based on rectangular window function is employed to emulate unknown time-varying noise. Artificial measurement noise following this characteristic is injected into the navigation sensor output signal during predefined time segments to simulate scenarios where sensors experience abnormal environmental interference. The MNCN based on the rectangular window function varies slowly according to the following equation

(62) $$R_{s,k}^{i}={\left(1+50\alpha_{s,k}\right)}^{2}{\tilde{R}}_{s}^{i}$$

(63) $$\alpha_{s,k}=\tanh\left(0.1T_{s}\left(t_{k}-t_{\mathrm{start}}\right)\right)+\tanh\left(0.1T_{s}\left(t_{\mathrm{end}}-t_{k}\right)\right)$$

where $T_{s}$ is the output period of sensor *s*, $t_{k}$ is the time corresponding to time step *k*, and $t_{start}$ and $t_{end}$ denote the start time and the end time of noise characteristic variation segment, respectively.

**Figure 3:**
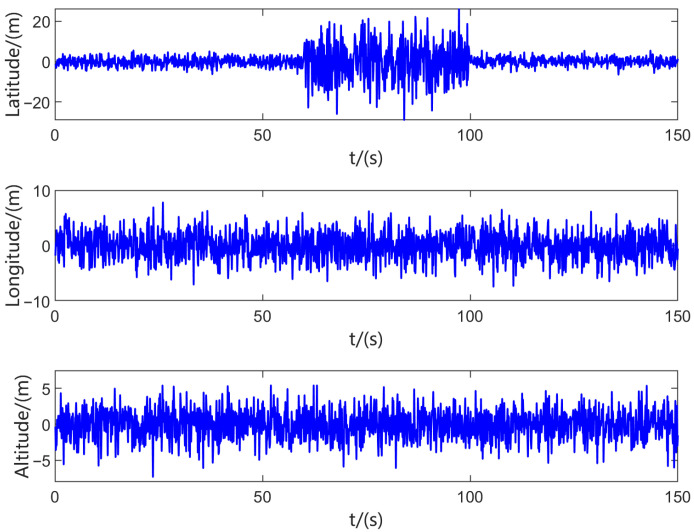
Position measurement noise of GNSS.

Assuming that the latitude channel of GNSS during 60–100 s exhibits the above-mentioned noise variation characteristics, the position measurement noise of GNSS is shown in Figure 3.

As illustrated in Figure 3, during the period from 60 s to 100 s, the measurement noise characteristics of the GNSS latitude channel no longer follow a Gaussian distribution with a variance of ${\tilde{R}}_{s}^{i}$, and exhibit significant differences compared to the noise characteristics of other time periods in the same channel and those of normally functioning channels. If abnormal sensor measurements enter the information fusion module and participate in the filtering process, the standard KF algorithms will result in reduced accuracy of the relevant local filters, ultimately degrading the performance of the navigation system. Therefore, the IVBAFKF is proposed in this paper to maintain the filtering accuracy of the navigation system during sensor anomalies.

Subsequently, the critical parameters governing the adaptive adjustment mechanism of the forgetting factor model are determined. In practice, the values of these parameters can be determined within an empirically established range. To enhance the adaptive capability, these parameters can be further optimized by leveraging the results of simulation experiments.

**Figure 4:**
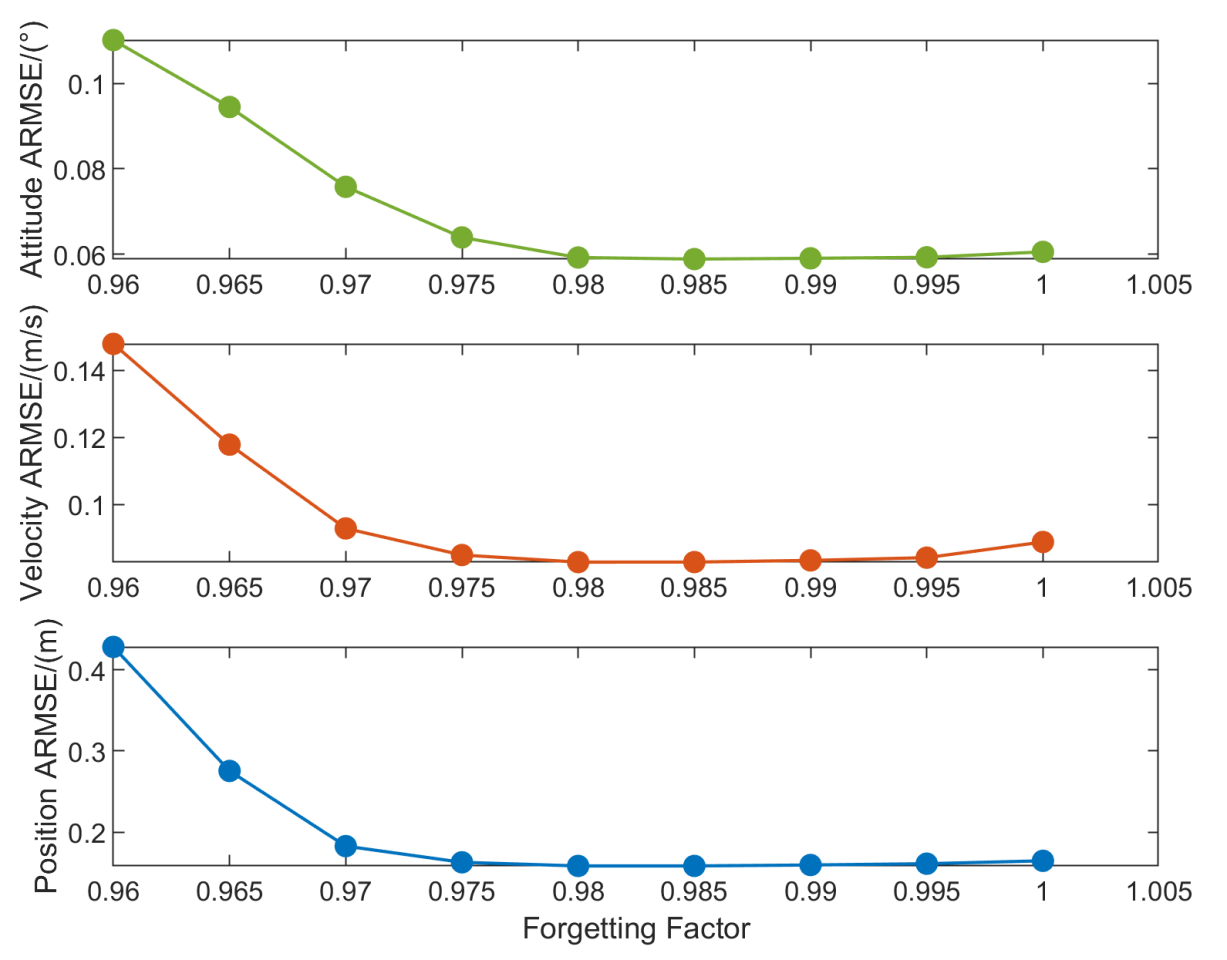
The ARMSEs of estimation results under different forgetting factors.

The ARMSEs of navigation states shown in Figure 4 are obtained based on Monte Carlo simulations under different values of the forgetting factor ρ, where the tuning parameter τ is set to 12 and the number of iterations *N* is set to 10. As illustrated in Figure 4, when the forgetting factor ρ is below 0.98, the ARMSE exhibits a decreasing trend with increasing ρ. Within the range of 0.98 to 0.995, the ARMSE remains relatively stable. A higher forgetting factor ρ enhances system stability and mitigates divergence, whereas optimizing its value within a judicious range improves robustness against measurement noise. Therefore, ρ can be set to 0.995 in VBAFKF to achieve the balance between these competing objectives. For ρ exceeding 0.995, the ARMSE shows a slight increase yet stays at a relatively low level. Consequently, an ideal range for ρ is set as $\left[0.98,1\right]$, with the corresponding upper and lower bound parameters set as $l_{1}=0.98$ and $l_{3}=0.02$.

**Figure 5:**
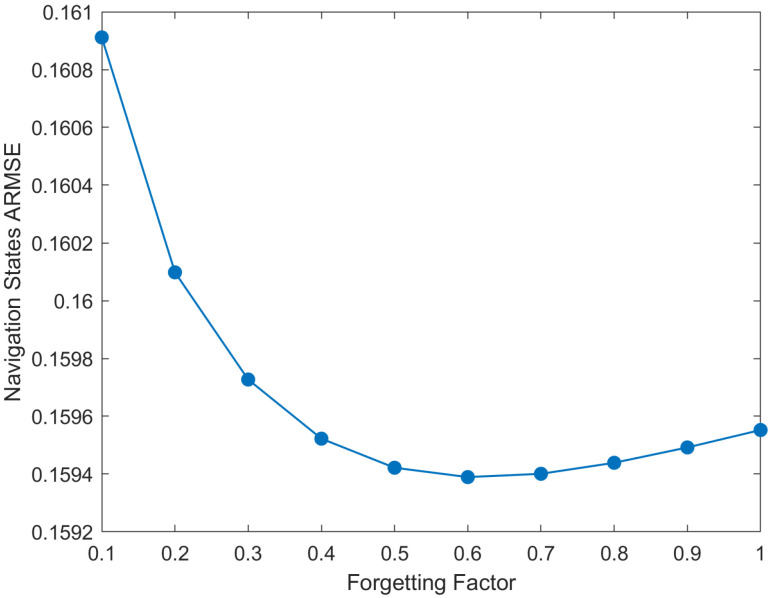
The mean ARMSEs of estimation results with different $l_{2}$.

The mean ARMSEs of estimation results with different $l_{2}$ values are shown in Figure 5. As illustrated in Figure 5, the mean ARMSE initially decreases rapidly and subsequently increases slightly with the rise of $l_{2}$. The minimum mean ARMSE occurs at $l_{2}=0.6$, where the estimation results achieve optimal accuracy. Consequently, $l_{2}=0.6$ is selected for subsequent AKF simulations.

To evaluate the effectiveness of the proposed algorithm in handling anomalous measurements, comparative simulations are conducted separately for each type of anomaly using the noise injection parameters specified in Table 2. The investigated algorithms include (1) KF based on true covariance matrices (KFTCM), (2) KF based on nominal covariance matrices (KFNCM), (3) Sage–Husa AKF, (4) Adaptive Robust Filtering (ARF), (5) FKF based on TCM (FKFTCM), (6) FKF based on NCM (FKFNCM), (7) FKFTCM with ISF adjustment, (8) FKFNCM with ISF adjustment, (9) VBAFKF (ρ=0.995), (10) IVBAFKF.

The ISFs are assigned a value of $\beta_{a}=\beta_{v}=\beta_{p}=\beta_{m}=0.25$ in FKF, and are adjusted adaptively according to the following equation:

(64) $$\beta_{k}^{i}=\frac{∥{\left(P_{k}^{i}\right)}^{-1}∥}{\sum_{j=m,a,v,p}∥{\left(P_{k}^{j}\right)}^{-1}∥}$$

**Figure 6:**
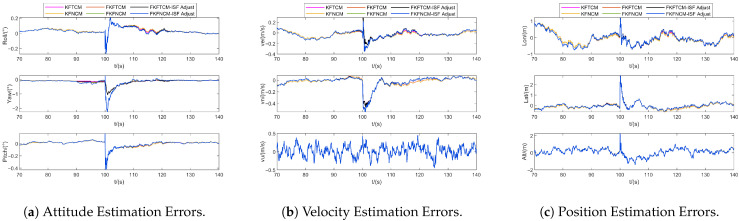
The estimation errors of navigation states (Noise ID: 4).

Within the adaptive federated filtering framework, the two core mechanisms are the ISF adjustment and adaptive local filters. Taking Noise ID 4 as a case study, Figure 6 compares the performance of centralized and federated filtering algorithms, along with the impacts of ISF adjustment on estimation accuracy.

It can be seen from Figure 6 that under ideal conditions with true covariance matrices, the estimation accuracy of FKFTCM is comparable to that of KFTCM, and similarly for FKFNCM versus KFNCM, indicating that the system structure has a limited impact on filtering performance. Additionally, due to the periodic reset operation of the federated filter, under the current system architecture, the regulatory effect of the ISF on its overall performance is constrained, making it difficult for the ISF-based adaptive mechanism to effectively compensate for estimation deviations caused by measurement noise mismatch. Therefore, subsequent simulations focus on the mechanisms of adaptive local filter to address the issue of measurement noise mismatch.

**Figure 7:**
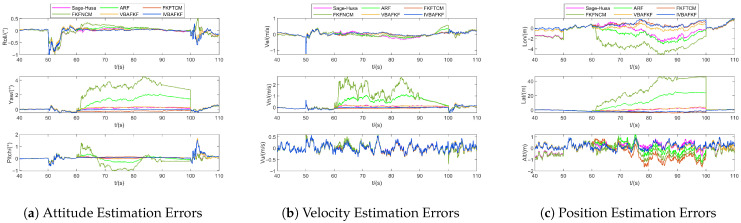
The estimation errors of LFv (Noise ID: 1).

In the following, taking Noise ID 1 as a case study, anomalous measurement noise is introduced into the north velocity channel of GNSS. Figure 7 depicts the estimation errors of velocity-based local filter (LFv) obtained by different FKF-based algorithms. Estimation errors of other local filters are omitted here for conciseness, as their responses to the velocity noise anomaly are relatively minor and not visually salient. As shown in Figure 7, all adaptive filtering algorithms improve the estimation accuracy of the navigation states to varying degrees compared to FKFNCM. Among them, IVBAFKF exhibits relatively superior performance, with the mean errors in attitude, velocity, and position being close to zero and the smallest fluctuation range, validating the effectiveness of IVBAFKF in addressing measurement noise mismatch.

**Figure 8:**
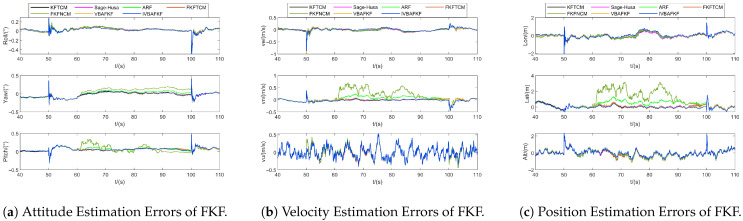
The navigation estimation errors of FKF (Noise ID: 1).

**Figure 9:**
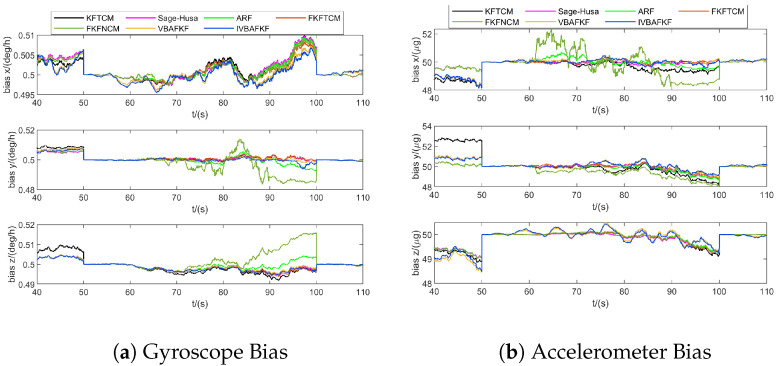
The IMU estimation errors of FKF (Noise ID: 1).

Figure 8 and Figure 9 show the estimation results of navigation states and bias of IMU sensors obtained by different FKF-based algorithms. The results shown in Figure 8 indicate that the estimation errors of FKFNCM exhibits noticeable anomalies due to the variations in the measurement noise characteristics during 60–90 s, while both VBAFKF and IVBAFKF exhibit reduced estimation errors to some extent. Moreover, IVBAFKF further enhances the estimation accuracy compared to VBAFKF, which demonstrates its superior tracking capability for the time-varying MNCM in dynamic environments. Figure 9 shows that compared to other adaptive filtering algorithms, IVBAFKF yields an improvement in the estimation of IMU errors, resulting in enhanced accuracy.

The ARMSEs of navigation states during the stage of measurement noise anomalies, obtained from 30 Monte Carlo simulations, are listed in Table 3. In addition, the Mean Relative Error Reduction Percentage (MRERP) listed is a normalized metric quantifying the relative performance disparities between different algorithms and FKFNCM.

Analysis of the data in Table 3 reveals the following:

1. The FKFNCM performs measurement updates based on a nominal MNCM. When the actual noise statistics change, these fixed parameters cannot accurately reflect the variations. Consequently, the ARMSE of the navigation state estimates increases significantly compared to FKFTCM. Under Noise ID 2, the position estimation error reaches 3.9634 m, exposing the inadequacy of the fixed noise model in adapting to dynamic environments.
2. Based on the IW distribution assumption, VBAFKF dynamically estimates the MNCM using a VBAKF as local filter with a constant forgetting factor. Experimental data show that this algorithm reduces the average ARMSE of navigation states by 41.99% across the four noise scenarios, effectively mitigating the impact of abnormal noise characteristics on filtering accuracy.
3. By adaptively adjusting the forgetting factor, IVBAFKF achieves higher accurate in tracking MNCM variations. Across all the current test scenarios, it demonstrates superior navigation state estimation accuracy over the VBAFKF, with an average improvement of 1.22%. IVBAFKF exhibits enhanced robustness in dynamic noise environments with time-varying statistical properties.
4. Compared to traditional adaptive filter algorithms such as Sage–Husa and ARF, IVBAFKF comprehensively outperforms them in the MRERP, achieving an average relative error reduction percentage of 43.21%, significantly higher than Sage–Husa (33.54%) and ARF (28.31%). This establishes its performance advantage in handling complex noise environments.

These quantitative analysis results collectively validate the effectiveness of IVBAFKF in accurately tracking noise statistic through its adaptive adjustment mechanism, providing a reliable solution for high-precision navigation state estimation in dynamic environments.

The limitations of the proposed method are mainly reflected in the following two aspects: (1) the effectiveness of the adaptive ISF adjustment mechanism are limited under the current federated filter architecture with periodic reset operations to enhance the system’s fault tolerance, hindering fully compensate for estimation deviations caused by measurement noise mismatch; (2) the IVBAFKF may employ empirical parameters in adaptive forgetting factor model to obtain adaptability to measurement noise mismatch. However, it still requires parameter tuning to achieve optimal results and its performance is somewhat contingent upon accurate prior knowledge of the system’s noise statistics.

## 5. Conclusions

This paper proposes a multi-sensor asynchronous navigation information fusion method based on an adaptive federated filtering frame. To address the accuracy degradation caused by time-varying measurement noise with uncertain statistics, an improved VBAKF is developed and embedded as local filter, which adaptively adjusts the forgetting factor based on the SMD of the measurement innovation. The effectiveness of the proposed algorithm is validated though a comparative study based on Monte Carlo simulations under complex measurement noise conditions. Consequently, the simulation results support the following conclusions:

1. The proposed adaptive forgetting factor strategy significantly enhances tracking accuracy of the MNCM variations and maintains robustness under deteriorated measurement conditions induced by increased noise levels or undetectable minor sensor faults.
2. The navigation accuracy of the proposed algorithm is improved compared to baseline FKF algorithms (e.g., FKFTCM, FKFNCM, and VBAFKF), as demonstrated by performance metrics of ARMSE. The proposed algorithm achieves an average reduction of 43.21% in the ARMSEs of navigation states compared to FKFNCM.

The proposed navigation information fusion approach provides a systematic solution for fusing multi-source asynchronous navigation data under varying sensor configurations, and exhibits a certain degree of robustness against time-varying measurement noise with non-Gaussian statistic.

To address the challenges in real-world scenarios where prior information is insufficient or unavailable, future research will focus on two main directions to enhance the proposed navigation fusion scheme: (1) further optimization of the adaptive forgetting factor mechanism by designing more sophisticated mapping functions and adjustment strategies, thereby improving its responsiveness to complex noise variations; and (2) development of an intelligent parameter-tuning framework that leverages optimization algorithms (e.g., Particle Swarm Optimization and Genetic Algorithm) to automatically identify optimal parameter configurations. These efforts aim to reduce reliance on empirical methods and improve the scheme’s practicality in real-world applications. Ultimately, these research directions are designed to systematically extend the core methodology proposed in this paper while addressing its limitations. 

## Figures and Tables

**Table 1:** Sensor parameters of the simulated integrated navigation system.

Sensor	Parameter	Value	Sampling Rate
IMU	Gyro Constant Drift	0.5°/h	50 Hz
	Gyro Angular Random Walk	0.15° $/\sqrt{h}$	
	Accelerometer Bias	50 µg	
	Accelerometer Noise	10 µg/$\sqrt{\mathrm{Hz}}$	
GNSS	Position Noise	2 m $\left(1\sigma \right)$	10 Hz
	Velocity Noise	1 m/s $\left(1\sigma \right)$	
Odometer	Velocity Noise	1 m/s $\left(1\sigma \right)$	10 Hz
Magnetometer	Attitude Noise	1° $\left(1\sigma \right)$	50 Hz

**Table 2:** Injection parameters of anomalous measurement noise.

Noise ID	Sensor	Channel	Time Range
1	GNSS	North Velocity	60–90 s
2	GNSS	Longitude	120–150 s
3	Odometer	Forward Velocity	80–110 s
4	Magnetometer	Yaw	90–120 s

**Table 3:** Performance comparison of different algorithms.

Noise ID	Algorithm	Attitude (°)	ARMSE Velocity (m/s)	Position (m)	Mean
1	KFTCM	0.0998	0.1667	0.5337	–
	FKFTCM	0.0998	0.1680	0.5396	–
	FKFNCM	0.1456	0.3466	1.5520	–
	Sage–Husa	0.0998	0.1705	0.5493	–
	ARF	0.1029	0.1867	0.6593	–
	VBAFKF	0.0995	0.1655	0.4906	–
	IVBAFKF	0.0991	0.1639	0.4826	–
2	KFTCM	0.1043	0.1611	0.7280	–
	FKFTCM	0.1063	0.1624	0.8434	–
	FKFNCM	0.1535	0.4441	3.9634	–
	Sage–Husa	0.1070	0.1950	2.5923	–
	ARF	0.1157	0.2685	3.1427	–
	VBAFKF	0.1060	0.1610	1.0996	–
	IVBAFKF	0.1045	0.1564	0.7590	–
3	KFTCM	0.1622	0.5520	1.1926	–
	FKFTCM	0.1666	0.5676	1.3010	–
	FKFNCM	0.1680	0.7760	1.5553	–
	Sage–Husa	0.1419	0.2220	0.6876	–
	ARF	0.1433	0.3214	0.7853	–
	VBAFKF	0.1410	0.2160	0.6645	–
	IVBAFKF	0.1411	0.2073	0.6559	–
4	KFTCM	0.1898	0.1929	0.6769	–
	FKFTCM	0.1911	0.1933	0.6774	–
	FKFNCM	0.2830	0.1943	0.6774	–
	Sage–Husa	0.3037	0.1953	0.6788	–
	ARF	0.2831	0.1954	0.6787	–
	VBAFKF	0.1959	0.1866	0.6477	–
	IVBAFKF	0.1935	0.1861	0.6470	–
MRERP	Sage–Husa	17.48%	44.44%	38.69%	33.54%
	ARF	17.14%	35.92%	31.89%	28.31%
	VBAFKF	27.36%	48.03%	50.58%	41.99%
	IVBAFKF	27.86%	48.76%	53.02%	43.21%

The symbol “–” denotes that the data does not exist.

## Data Availability

The datasets generated during and/or analyzed during the current study are available from the corresponding author on reasonable request.
